# Supplementary material for: Hepatocyte Specific gp130 Signalling Underlies APAP Induced Liver Injury
Source: Int J Mol Sci. 2022 Jun 25;23(13):7089. doi: 10.3390/ijms23137089 (PMC9266364; doi:10.3390/ijms23137089)
Supplement: Supplementary file 1 [file ijms-23-07089-s001.zip › ijms-1777362-supplementary.pdf]

International Journal of
Molecular Sciences

MDPI

Supplementary figures

Hepatocyte Specific gp130 Signalling Underlies APAP Induced Liver Injury

Jinrui Dong [1], Wei-Wen Lim [1,2], Shamini G. Shekeran [1], Jessie Tan [2], Sze Yun Lim [1], Joyce Wei Ting Goh [1], Benjamin L. George [1], Sebastian Schafer [1], Stuart A. Cook [1,2,3,*] and Anissa A. Widjaja [1,*]

[1] Cardiovascular and Metabolic Disorders Program, Duke-National University of Singapore Medical School, Singapore 169857, Singapore; djrnku@gmail.com (J.D.); lim.wei.wen@nhcs.com.sg (W.-W.L.); shamini_g@duke-nus.edu.sg (S.G.S.); sy76@nus.edu.sg (S.Y.L.); joyce.goh@duke-nus.edu.sg (J.W.T.G.); ben.george@duke-nus.edu.sg (B.L.G.); sebastian@duke-nus.edu.sg (S.S.)

[2] National Heart Research Institute Singapore, National Heart Centre Singapore, Singapore 169857, Singapore; gmstje@nus.edu.sg

[3] MRC-London Institute of Medical Sciences, Hammersmith Hospital Campus, London W12 0NN, UK

* Correspondence: stuart.cook@duke-nus.edu.sg (S.A.C.); anissa.widjaja@duke-nus.edu.sg (A.A.W.)

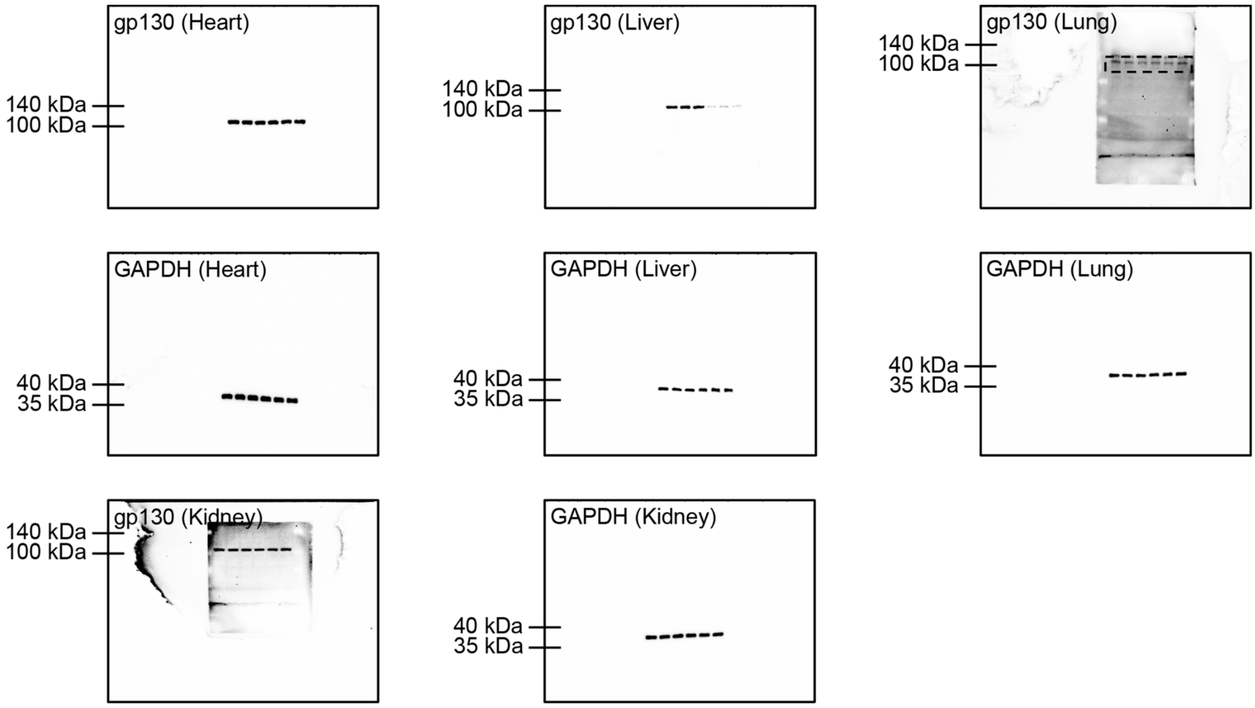

Figure S1. Uncropped blot images for Western blots data shown in Figure 1. Uncropped blots for gp130 and GAPDH expression in the heart, liver, lung and kidney tissue from WT and CKO^{gp130} mice (n = 3/genotype).

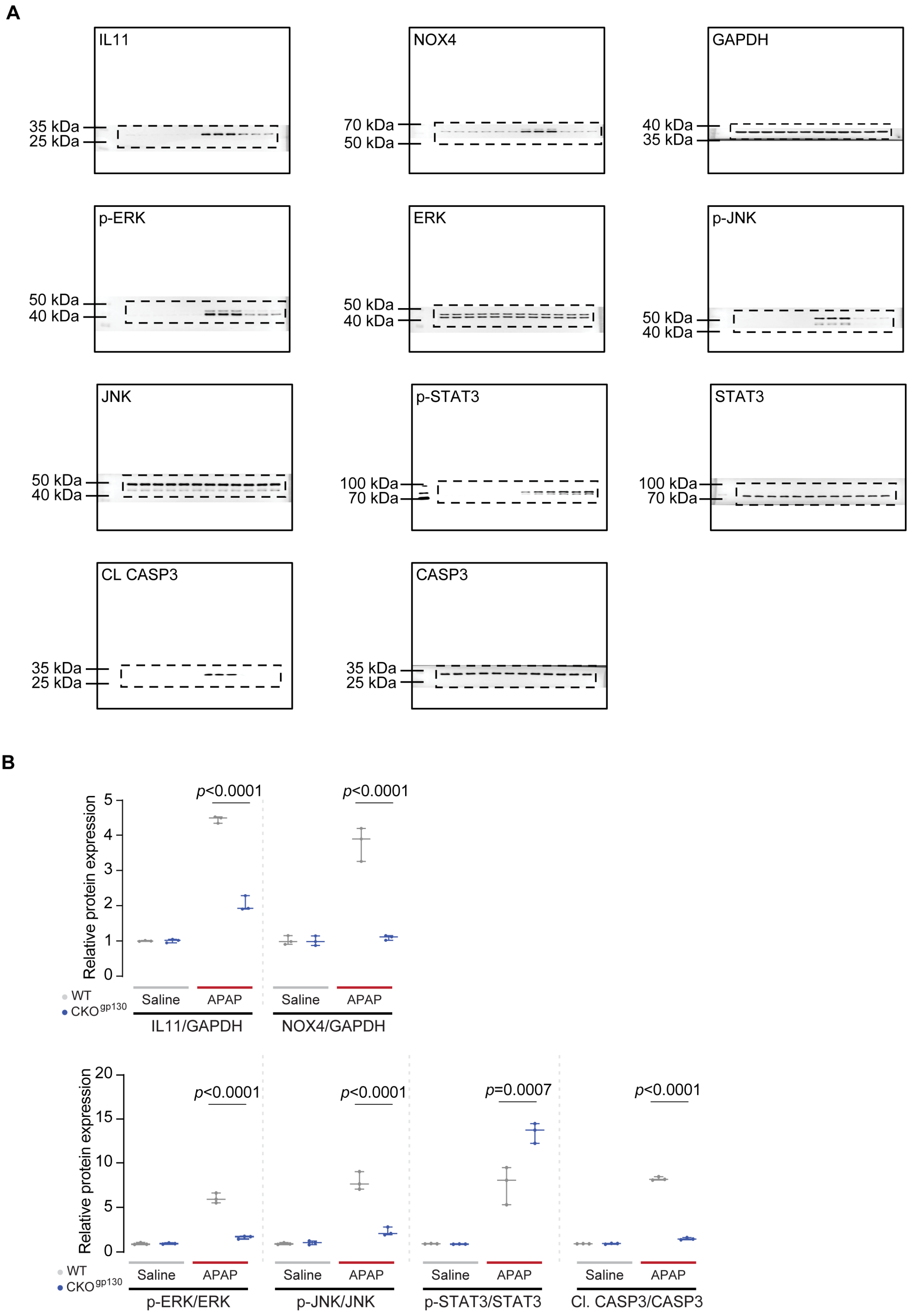

Figure S2. Uncropped blot images and densitometry analyses for Western blots data shown in Figure 2. **(A)** Uncropped blots and **(B)** densitometry analyses for hepatic expression of IL11, NOX4, GAPDH, p-ERK, ERK, p-JNK, JNK, p-STAT3, STAT3, Cl. CASP3, and CASP3 (n=3/group) from saline or APAP-injected WT and CKO^{gp130} mice (n = 3/group)..

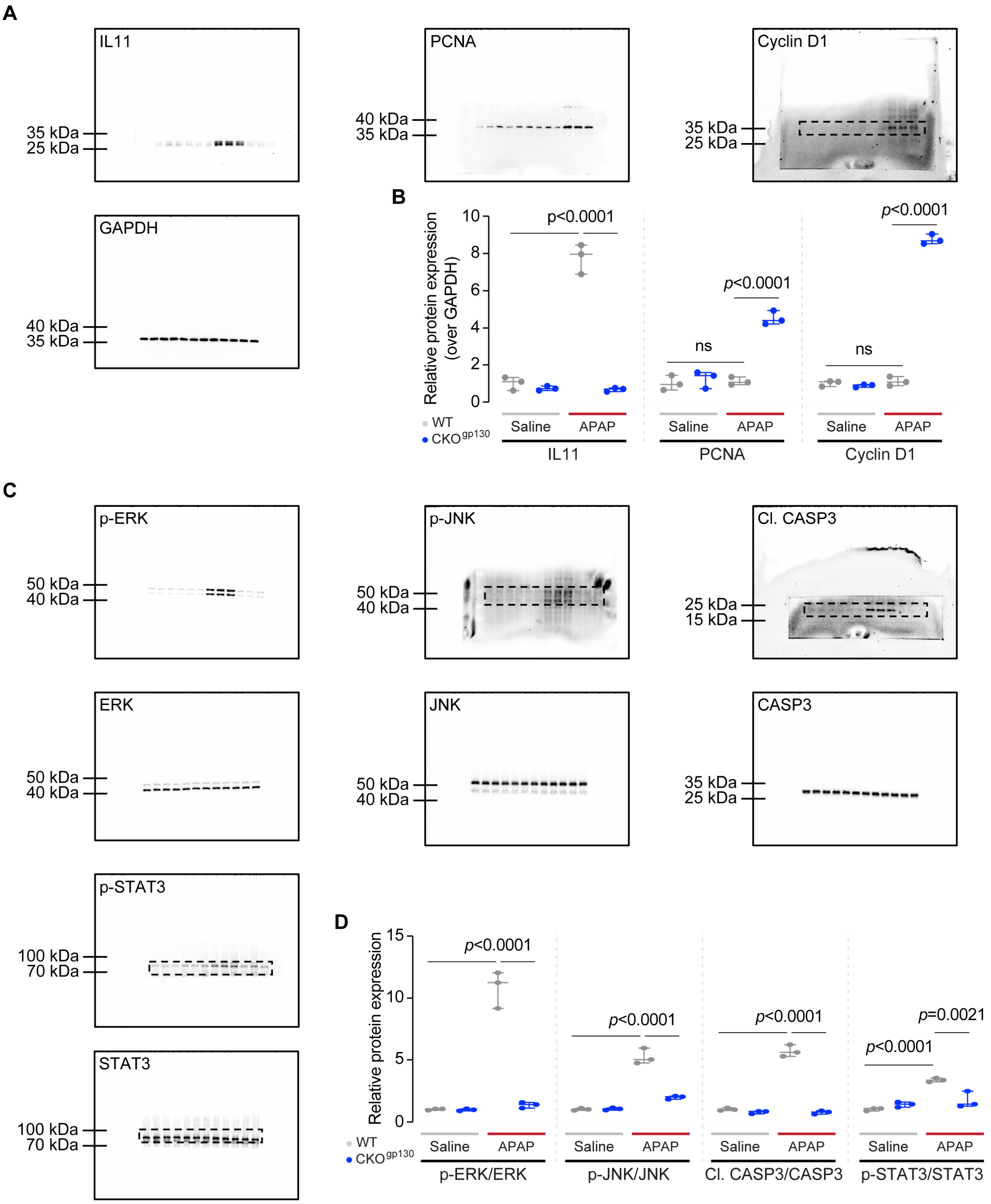

Figure S3. Uncropped blot images and densitometry analyses for Western blots data shown in Figure 3. **(A)** Uncropped Western blot images and **(B)** densitometry analyses showing hepatic levels of IL11, PCNA, Cyclin D1, and GAPDH as internal control, **(C)** uncropped Western blot images and **(D)** densitometry analyses of hepatic p-ERK, ERK, p-JNK, JNK, Cl. CASP3, CASP3, p-STAT3, and STAT3 in WT and CKO^{gp130} mice. **(B, D)** Data are shown as box-and-whisker with median (middle line), 25th–75th percentiles (box) and min-max values (whiskers); 2-way ANOVA with Sidak's correction; n=3/group.

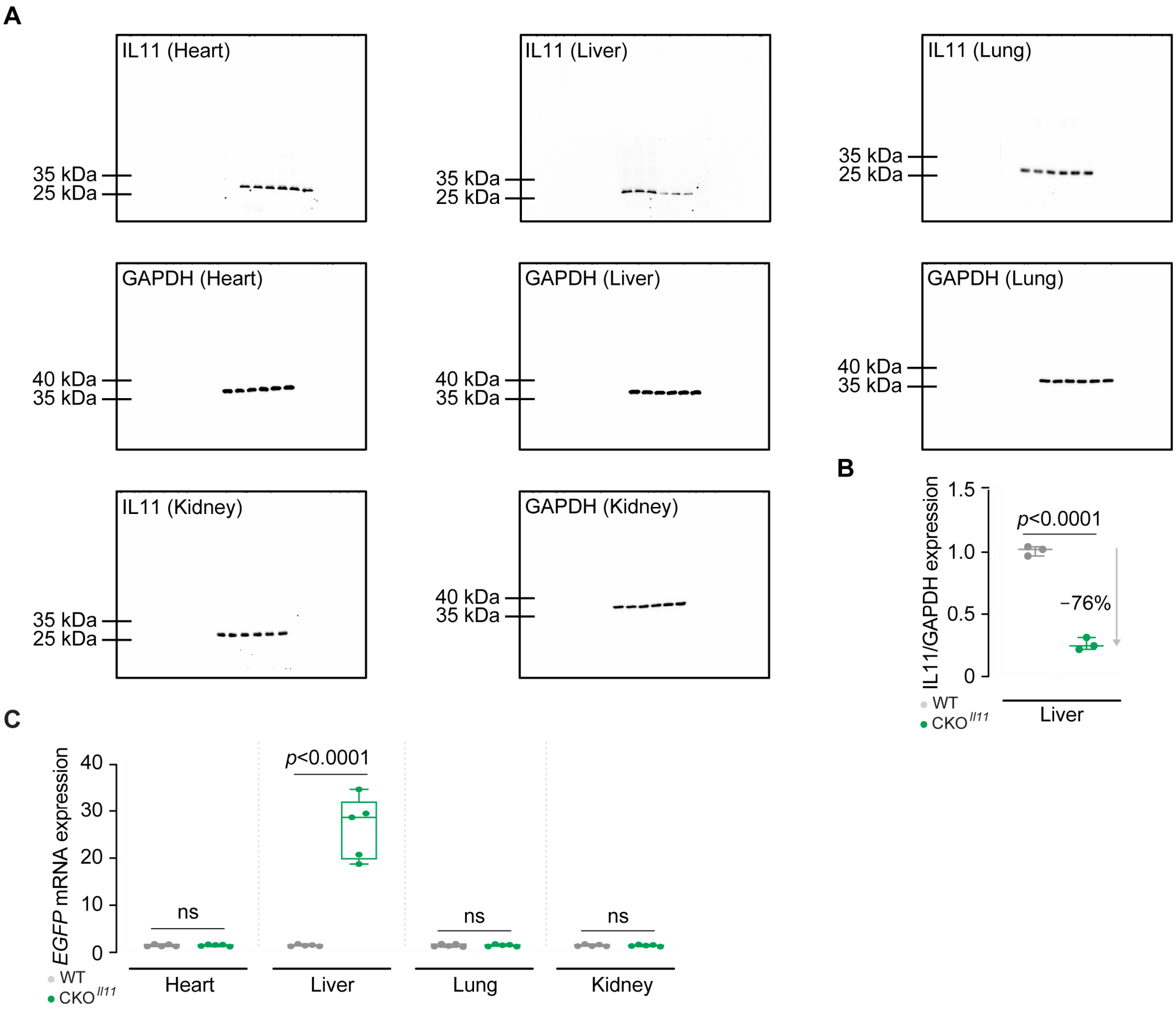

Figure S4. Generation and validation of hepatocyte-specific *Il11*-deficient mice. **(A)** Uncropped Western blot images of IL11 and GAPDH in the heart, liver, lung and kidney tissue (n=3/genotype), **(B)** densitometry analyses of IL11 expression in the livers (n=3/group), and **(C)** hepatic GFP mRNA expression in the heart, liver, lung and kidney (n=5/group) isolated from WT and CKO^{Il11} mice **(B-C)** Data are shown as box-and-whisker with median (middle line), 25th–75th percentiles (box), and minimum-maximum values (whiskers); 2-tailed Student's *t*-test.

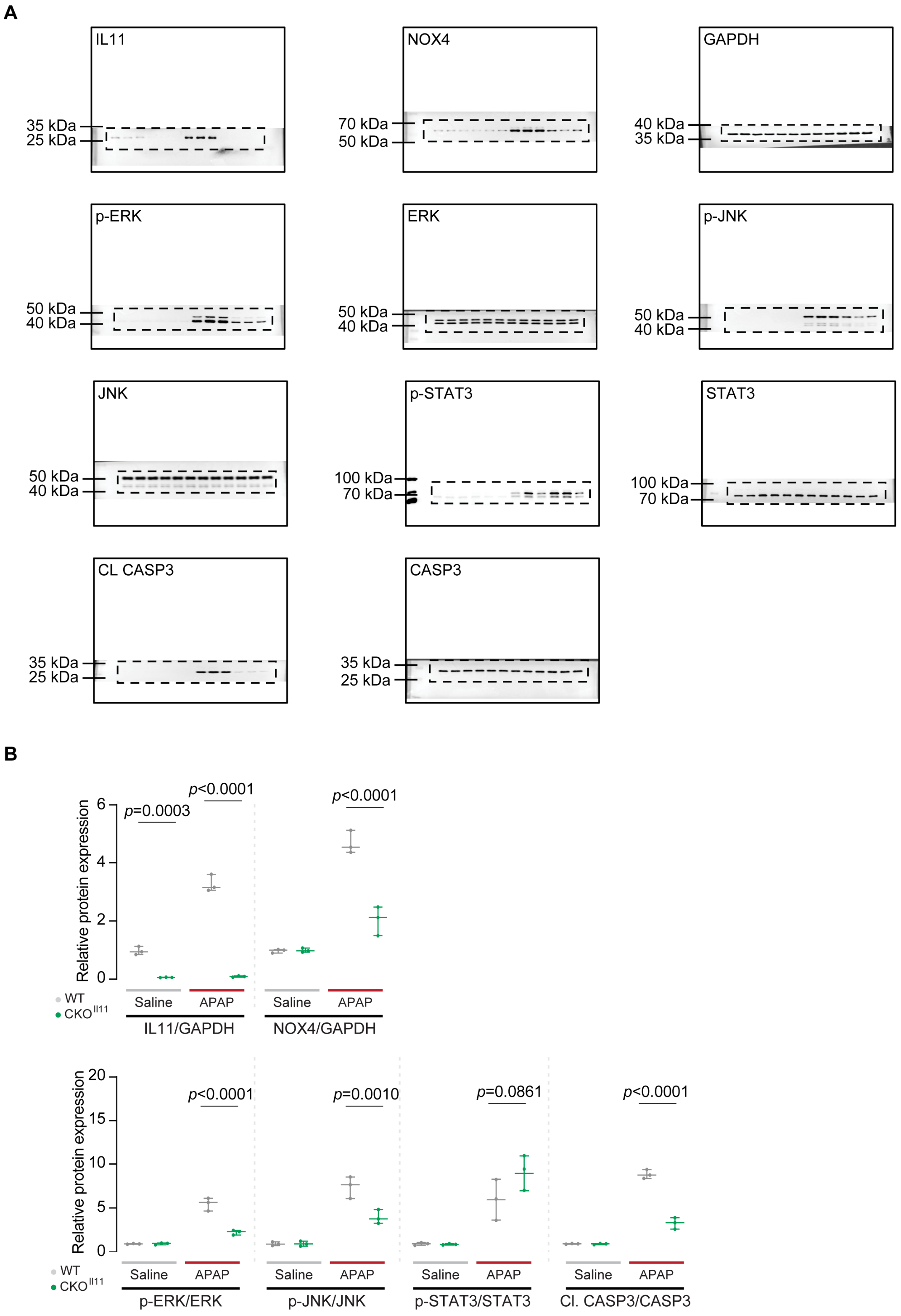

Figure S5. Uncropped blot images and densitometry analyses for Western blots data shown in Figure 5. **(A)** Uncropped blots and **(B)** densitometry analyses for hepatic expression of IL11, NOX4, GAPDH, p-ERK, ERK, p-JNK, JNK, p-STAT3, STAT3, Cl. CASP3, and CASP3 (n=3/group) from saline or APAP-injected WT and CKO^{Il11} mice (n=3/group).

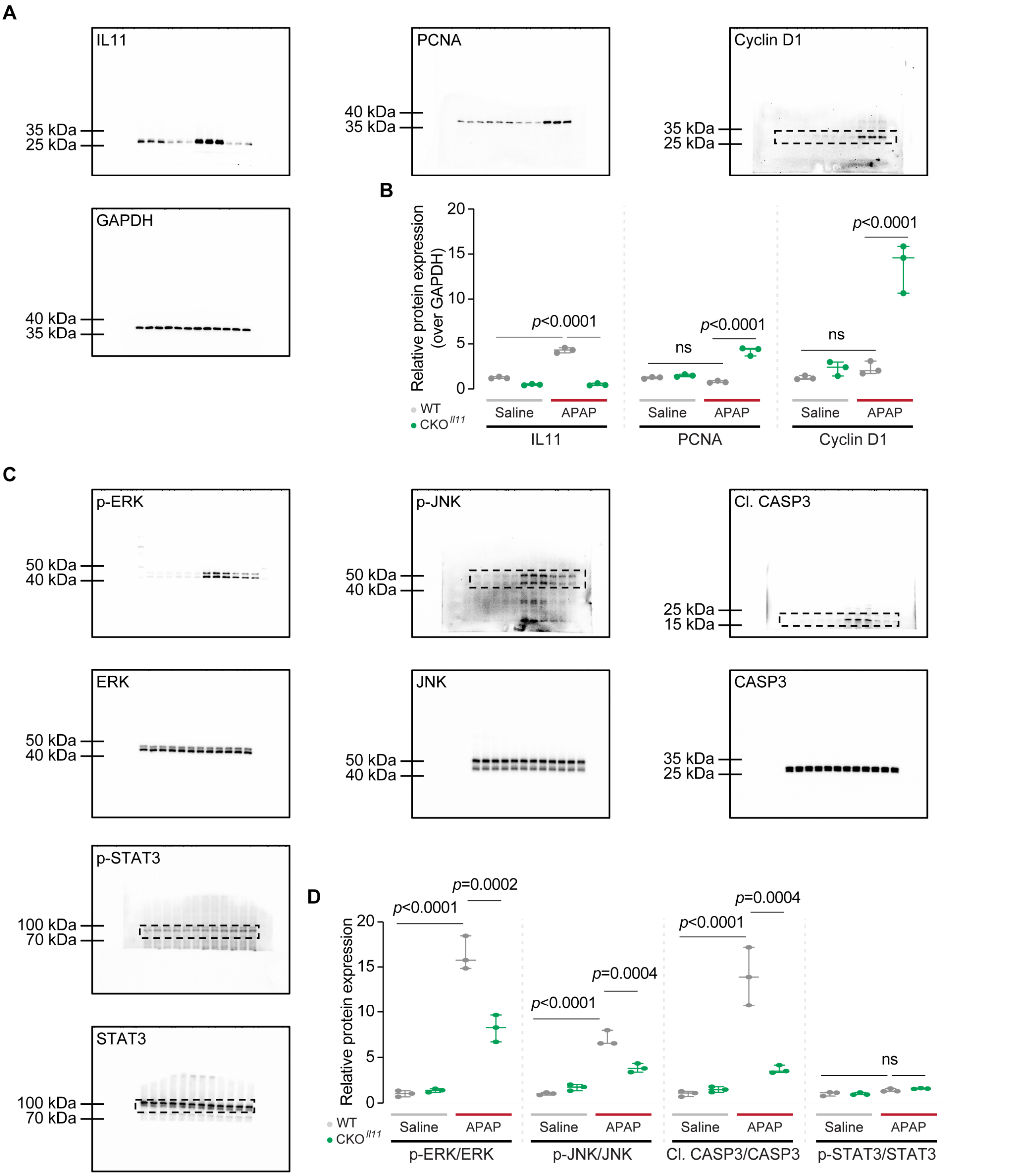

Figure S6. Uncropped blot images and densitometry analyses for Western blots data shown in Figure 6. **(A)** Uncropped Western blot images and **(B)** densitometry analyses showing hepatic levels of IL11, PCNA, Cyclin D1, and GAPDH as internal control, **(C)** uncropped Western blot images and **(D)** densitometry analyses of hepatic p-ERK, ERK, p-JNK, JNK, Cl. CASP3, CASP3, p-STAT3, and STAT3 in WT and CKO^{Il11} mice. **(B, D)** Data are shown as box-and-whisker with median (middle line), 25th–75th percentiles (box) and min-max values (whiskers); 2-way ANOVA with Sidak's correction; n=3/group.
